# Maternal microbe-specific modulation of the offspring microbiome and development during pregnancy and lactation

**DOI:** 10.1080/19490976.2023.2206505

**Published:** 2023-05-15

**Authors:** Min Tian, Qihui Li, Tenghui Zheng, Siwang Yang, Fang Chen, Wutai Guan, Shihai Zhang

**Affiliations:** a Guangdong Province Key Laboratory of Animal Nutrition Control, College of Animal Science, South China Agricultural University, Guangzhou, China; b College of Animal Science and National Engineering Research Center for Breeding Swine Industry, South China Agricultural University, Guangzhou, China; c Department of Molecular Biology, University of Texas Southwestern Medical Center, Dallas, TX, USA

**Keywords:** Maternal microbiota, microbial metabolite, fetus, neonate, immunity, brain development

## Abstract

The maternal microbiome is essential for the healthy growth and development of offspring and has long-term effects later in life. Recent advances indicate that the maternal microbiome begins to regulate fetal health and development during pregnancy. Furthermore, the maternal microbiome continues to affect early microbial colonization via birth and breastfeeding. Compelling evidence indicates that the maternal microbiome is involved in the regulation of immune and brain development and affects the risk of related diseases. Modulating offspring development by maternal diet and probiotic intervention during pregnancy and breastfeeding could be a promising therapy in the future. In this review, we summarize and discuss the current understanding of maternal microbiota development, perinatal microbial metabolite transfer, mother-to-infant microbial transmission during/after birth and its association with immune and brain development as well as corresponding diseases.

## GRAPHICAL ABSTRACT

**Figure:**
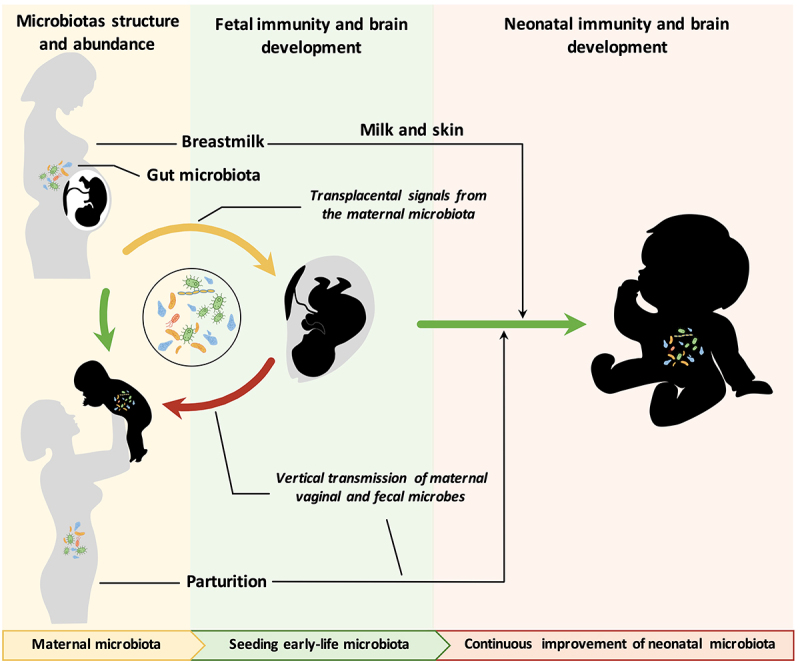

## 1. Introduction

The human body is colonized by hundreds of millions of commensal microbes, which are essential for maintaining the health of humans. Most of these microbes belong to four phyla: Firmicutes, Bacteroidetes, Actinobacteria and Proteobacteria. ^1,2^ In contrast to the relatively stable gut microbiota in adults, the neonatal microbiota undergoes dramatic fluctuation and exhibits high adaptability and plasticity. Recent advances in genome sequencing technologies have expanded our knowledge regarding microbiota colonization during early life and have helped decipher its critical functions in host physiological processes, such as metabolism,^3^ immunity,^4^ cognition^5^ and intestinal homeostasis.^6^ Disruption of microbiome establishment during the newborn period can lead to life-threatening diseases such as necrotizing enterocolitis (NEC)^7,8^ and late-onset sepsis (LOS)^9^ and might increase the risk of obesity,^10^ diabetes^11^ and asthma^12^ in adulthood.

Similar to the genome, the microbiome is inherited by the infant from the mother. The seeding of newborn intestinal bacteria is considered to largely occur during the birthing process. After birth, neonatal gut microbes are persistently affected by maternal factors via direct contact with the mother’s skin and breast milk. Full-term vaginal delivery and exclusive breastfeeding of neonates are considered the “golden rules” for optimal microbiota selection in neonates.^13^ Approximately 11% of maternal-derived bacterial strains (mainly *Bacteroides* and *Bifidobacterium*) persist during the first year of life.^14^ Since the neonatal microbiota is malleable in early-stage newborns, this period is a crucial time window for external intervention (such as with environmental and dietary factors) to regulate neonatal gut dysbiosis and its related diseases. This review focuses on the fluctuation of the maternal microbiome during pregnancy and lactation as well as the impact of the maternal microbiota and its metabolites on infants, including on early gut colonization, immune maturation, and brain development. Insights into the timing and mechanisms by which the maternal gut microbiota regulates the development of neonates can be used to develop gut microbiome-based therapies to prevent and/or treat related diseases in neonates.

## 2. Maternal microbiome and fetal health and development during gestation

### 2.1 Fluctuation of maternal gut microbes during pregnancy

**Figure 1.**
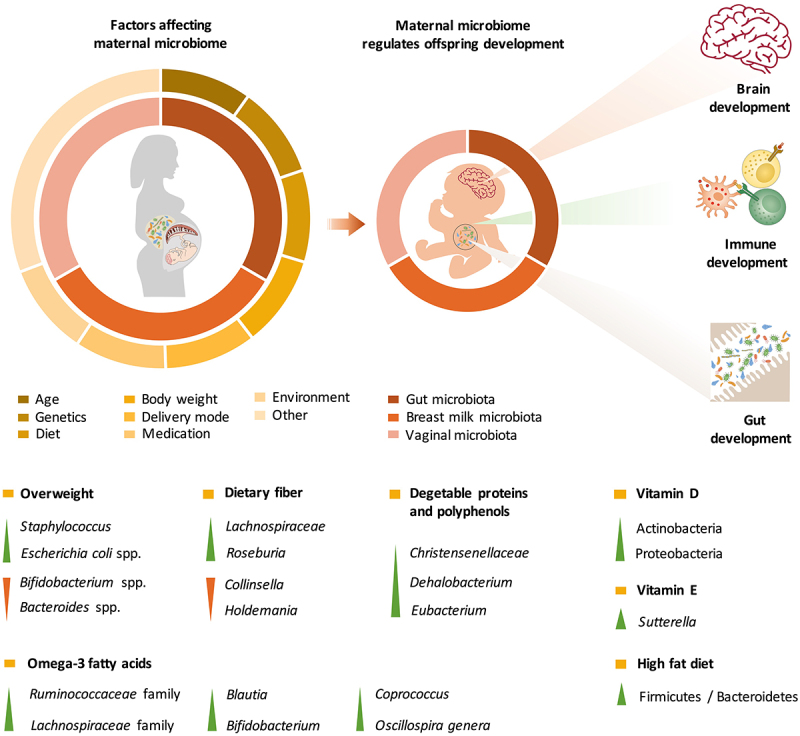
The maternal microbiota and its metabolites regulate fetal development before birth and during lactation. The maternal gut, breast milk and vaginal microbiota are major factors regulating offspring development. The maternal microbiome is influenced by a variety of factors, including age, genetics, diet, body weight, delivery mode, medication use, and the environment. The effects of various factors on the maternal microbiome may be passed on to the offspring through microbes, microbial products or metabolites, which regulate early gut colonization, immune maturation, and brain development in infants.

During pregnancy and lactation, maternal gut microbes support the increased energy demand for fetal growth and milk synthesis by digesting carbohydrates that are resistant to host-secreted digestive enzymes. Pregnancy involves hormonal, psychological and immunological changes that regulate gut microbiota variation during different stages of pregnancy. Prior studies have showen that *Proteobacteria* and *Actinobacteria* in the maternal gut are enriched during the third trimester of pregnancy, with increases in the levels of proinflammatory cytokines.^15^ However, recently, a comprehensive overview of the intestinal microbiota from approximately 1500 women indicated that the core microbiota of pregnant women and nonpregnant women was similar during the progression of gestation^16^ and remained stable during the perinatal period.^17^ Individual heterogeneity (including in body mass index (BMI), diet, antibiotic treatment, residency status and disease) is the dominant factor that shapes the intestinal microbiota during gestation (Figure 1).^16^

### 2.2 Influences on the maternal microbiome during pregnancy

#### 2.2.1 Maternal BMI

Overweight mothers undergo shifts in their intestinal microbiota during gestation, especially between the first and third trimesters.^18^ Decreased levels of *Bifidobacterium* spp. and *Bacteroides* spp. as well as increased levels of *Staphylococcus* and *Escherichia coli* spp. are commonly observed in overweight mothers.^19,20^ In addition, changes in gut microbes regulate maternal metabolism during pregnancy through their modification of metabolic hormones (such as adipokines, insulin and gastrointestinal polypeptides).^21^ Therefore, the microbial communities in overweight and obese pregnant women might induce inflammation, impair glucose and insulin tolerance, and further lead to gestational diabetes. Although maternal weight status has been found to affect the composition of the offspring microbiome in animal models,^22^ a meta-analysis of human studies found that maternal weight status had a limited predictive effect on the composition of the microbiome in infants.^23^

#### 2.2.2 Maternal dietary patterns

Recent evidence indicates that maternal dietary patterns and nutritional compounds are crucial factors that shape the intestinal microbial community (Figure 1). A higher Firmicutes/Bacteroidetes ratio in the gut during pregnancy was observed in mothers who consumed a high-fat diet.^24^ Dietary fiber is a predominant factor that regulates the richness and diversity of the gut microbiota.^25^ Pregnant women who are vegetarians exhibit decreases in the abundances of *Collinsella* and *Holdemania* and increases in the abundances of *Roseburia* and *Lachnospiraceae*, which are accompanied by an increased production of short-chain fatty acids (SCFAs).^26^ Conversely, deficiency in dietary fiber intake results in an increased abundance of *Collinsella* among the intestinal microbes and enhanced lactate fermentation in pregnant women.^27^ In addition to dietary fiber, plant-derived foods, such as vegetable proteins and polyphenols, increase the number of *Christensenellaceae, Dehalobacterium*, and *Eubacterium* ^28^ and the production of butyrate and propionate in the gut.^29,30^

Recent studies have also uncovered the effects of many other nutrients that shape the gut microbial community during pregnancy. For instance, a higher level of vitamin D intake has been found to decrease alpha diversity and lead to enrichment in *Actinobacteria* and *Proteobacteria* in pregnant women, which might result in inflammatory bowel disease, obesity, autism, allergy, and asthma.^31^ Lower vitamin E consumption is associated with higher levels of *Sutterella*,^31^ which can cause gastrointestinal disorders by degrading IgA.^32^ In addition, *Sutterella* has been found in greater abundance in infants with autism spectrum disorder.^33,34^ Omega-3 fatty acid intake increases the abundances of microbes in the *Ruminococcaceae* and *Lachnospiraceae* families as well as in the *Blautia, Bifidobacterium, Coprococcus*, and *Oscillospira* genera.^28^

An unhealthy maternal diets has been demonstrated to increase the risk of preterm delivery and impair embryonic growth and development.^35–37^ However, research on the effects of maternal diet composition on infant gut microbes is in the early stages. Consumption of a high-fat diet (more than 40% of total energy) during pregnancy decreases the number of *Bacteroides* species in the infant.^38^ However, the correlations between *Bacteroides* and the risk of developing obesity are still under intense debate.^39,40^ In addition, the influence of the mother’s dietary intake during pregnancy on *Prevotella* abundance is closely related to the risk of food allergy in the infant. The higher the abundance of *Prevotella* in the maternal gut is, the lower the risk of food allergy in infants at 12 months.^41^ Future studies need to clarify the optimal dietary formula for the establishment of healthy gut microbes during pregnancy.

#### 2.2.3 Maternal antibiotic exposure

To prevent preterm birth and infection after cesarean (C) section, approximately 40% of women are administered antibiotics during pregnancy.^42–44^ Broad-spectrum antibiotics, such as cephalosporins, are frequently prescribed to women in developed countries.^45,46^ Few recently published human studies have evaluated the effects of antibiotic treatment during pregnancy on the maternal microbiome. Maternal exposure to antibiotics during delivery can lead to a reduction in the infant’s gut microbial diversity.^23^ However, differences in the composition and diversity of the infant gut microbiome due to maternal antibiotic exposure have limited impact and may last only one month.^47,48^

### 2.3 Maternal microbiome and fetal health and development

#### 2.3.1 Maternal microbiome regulates fetal health during pregnancy

For over a century, the development of healthy infants was considered to occur in a sterile environment until birth through the vaginal canal.^49^ Unexpected microbial invasion is commonly associated with intrauterine infections and preterm labor.^50,51^ During early pregnancy, listeriosis (caused by *Listeria monocytogenes*) can disrupt the maturation of the fetal immune system and cause neonatal sepsis and death.^52,53^ Syphilis (caused by *Treponema pallidum* infection) leads to adverse pregnancy outcomes, and fetal transmission causes congenital diseases such as osteochondritis and meningitis in newborns.^54,55^ Although the pathophysiology regarding the transplacental transmission of pathogenic bacteria remains unknown, the impact of bacterial infections on congenital malformations and serious diseases in the fetus has been clearly shown.

**Figure 2.**
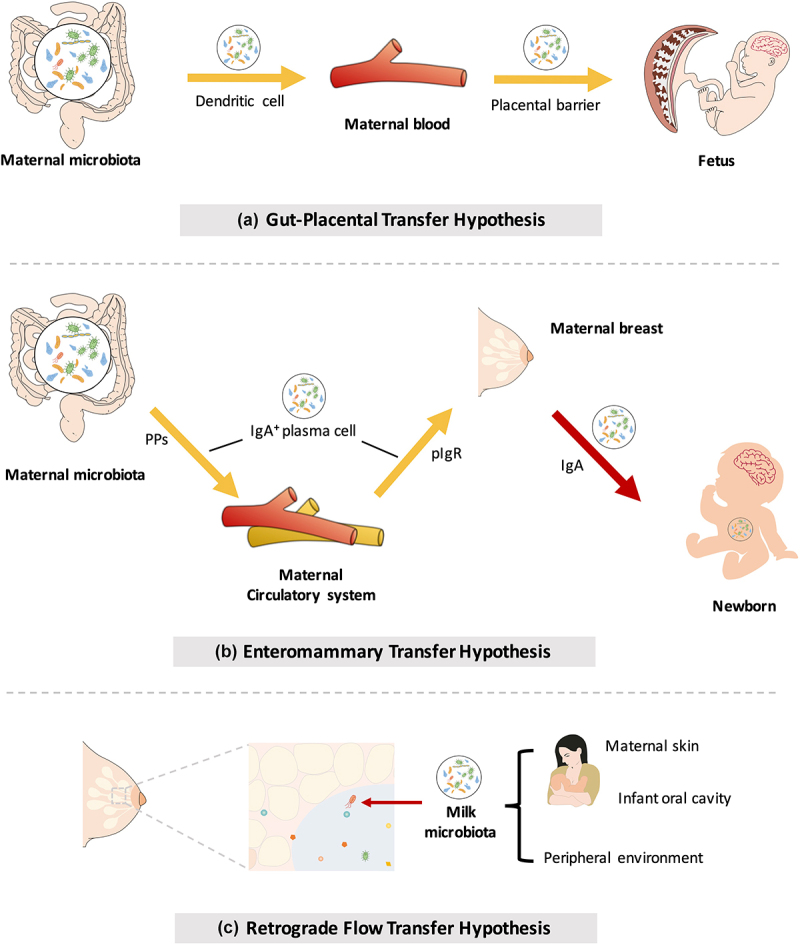
Mother-to-offspring bacterial transmission during pregnancy and lactation. (a) Gut-placental transmission hypothesis. This hypothesis states that transmission of the maternal microbiome to the fetus occurs before birth. Bacteria from the maternal intestine can be harvested by dendritic cells and then migrate to the fetus through the blood. (b) Enteromammary transfer hypothesis. Breast milk functions as the most important postnatal link between infants and mothers. In addition to macronutrients, micronutrients, oligosaccharides and immune factors, maternal milk contains a microbiota. This hypothesis states that the parent microbes combine with IgA in the gut to form a complex (IgA^+^). IgA^+^ plasma cells translocate to maternal mammary tissue via intestinal Peyer’s patches (PPs) to release microorganisms and secrete IgA. Breastfeeding newborns acquire nutrients and microorganisms in milk. Microorganisms in milk are transferred to colonize the neonatal gut. (c) Retrograde flow transfer hypothesis. This hypothesis proposes that the microbiota from the maternal skin, fetal oral cavity and environment enter the maternal mammary gland during lactation, thereby composing the milk microbial community.

Recently, with the development of 16S rRNA sequencing, this general dogma has been challenged. Bacterial signals have been detected in amniotic fluid,^56,57^ umbilical cord blood,^58^ and fetal membranes^59^ in babies who do not have infection or exhibit inflammation. These findings provide evidence for the transmission of the maternal microbiome to the fetus before birth. Bacteria from the maternal intestine can be harvested by dendritic cells and then migrate to the fetus through the blood (Figure 2). Although no consensus has been reached regarding the transfer of microbes before birth (discussed in Box 1), metabolites from the maternal microbiota are undoubtedly involved in the regulation of infant health and embryonic development and have long-term effects during postnatal life (Figure 1).^60,61^ The physiological mechanisms by which the maternal microbiome affects fetal immunity and brain development are now comparatively well established and are discussed below.

#### 2.3.2 Microbiota metabolites regulate fetal development during pregnancy

**Figure 3.**
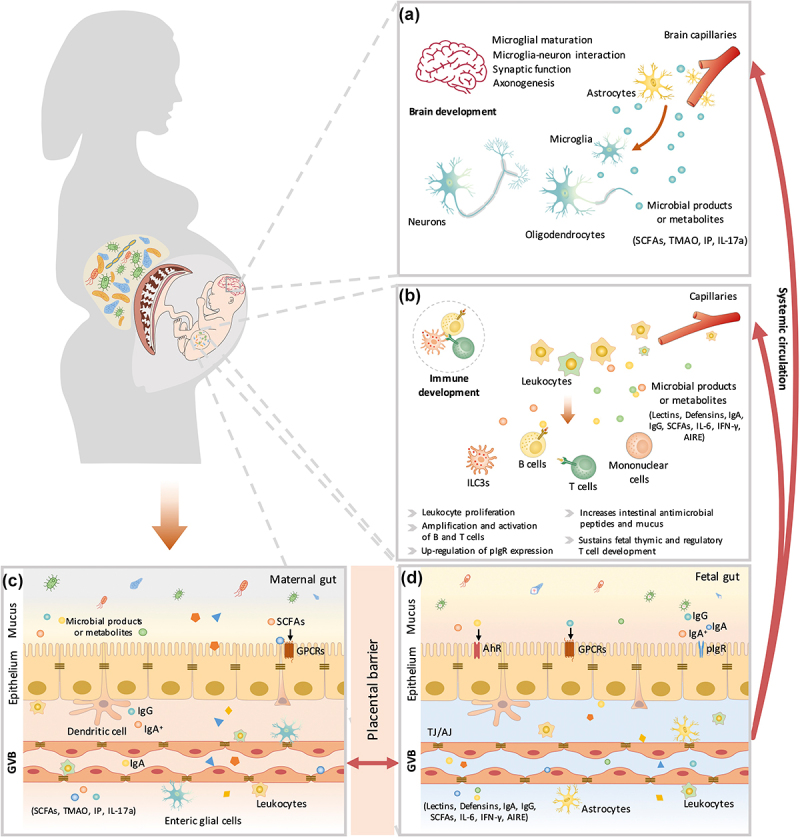
The maternal microbiota or microbiota metabolites regulate fetal immunity and brain development. Bacterial metabolites can be absorbed into the bloodstream and then transported to the fetus during placentation. These metabolites derived from maternal bacteria regulate multiple biological functions of the fetus. Short-chain fatty acids (SCFAs) produced by microbial fermentation of high-fiber diets may regulate microglial maturation, microglial cell-neuron interactions, and synaptic function in the hippocampus by activating fatty acid receptors (GPR41 and GPR43). During pregnancy, maternal microbes also increase axonal numbers and promote thalamocortical axon development in the fetal brain by modulating the levels of metabolites, such as trimethylamine-N-oxide (TMAO) and imidazole propionate (IP). In addition, pathogen infection during pregnancy increases proinflammatory cytokine IL-17a secretion, which is transmitted through the circulation, affects fetal brain development and induces abnormal cortical phenotypes. Maternal microbes are also involved in the regulation of fetal immune establishment during pregnancy. Maternal intestinal Escherichia coli HA107 colonization increases fetal intestinal leukocyte proliferation (that of type 3 innate lymphocytes (Ilc3s) and monocytes) as well as the production of intestinal antimicrobial peptides (C-type lectins and defensins of the REG family) and mucus. Some microbiota-derived compounds are microbial aryl hydrocarbon receptor (AhR) ligands, which promote the amplification of ILC3s. Maternal-derived SCFAs also regulate thymus and T-lymphocyte development. Maternal infection by a pathogen (Yersinia pseudotuberculosis) regulates fetal immunity through an increase in the levels of IL-6, which crosses the placental barrier and promotes intestinal Th17 cell responses and intestinal protective immunity in the fetus. Therefore, maternal microbial metabolites (AhR ligands and SCFAs) and the cytokine-mediated response (IL-6) synergistically regulate the early establishment of immunity before birth. AIRE, autoimmune regulator; AJ, adherens junction; GPCRs, G protein-coupled receptors; GVB, gut vascular barrier; IFN-γ, interferon γ; IgA, immunoglobulin A; IgA^+^, IgA^+^ plasma cells; IgG, immunoglobulin G; pIgr, polymeric immunoglobulin receptor; TJ, tight junction.

Metabolites from maternal bacteria can be absorbed into the bloodstream and then transported to the fetus through the placenta.^62^ These metabolites regulate multiple biological functions in the fetus, such as metabolic homeostasis.^63^ Recently, a growing amount of evidence has indicated that bacterially produced metabolites promote neural development and boost the immune system in the fetus (Figure 3).

##### 2.3.2.1 Brain development

Extremely premature birth (gestational age<28 weeks) is associated with high morbidity and incomplete neurodevelopment.^64^ The third trimester during pregnancy is the predominant period during which the maturation of the human brain and the establishment of cognitive capacities occur.^65^ Maternal obesity not only affects maternal microbes but also might be associated with neurodevelopmental disorders in offspring, such as deficits in cognition and sociality.^66^ A high-fiber diet alleviates maternal obesity-induced synaptic impairments in offspring by reshaping maternal gut microbes (increasing the abundances of *Bifidobacterium animalis*, *Prevotella*, and Clostridiales).^66^ The modification of microbes by a high-fiber diet is associated with the enrichment of SCFAs, which further regulate microglial maturation, microglial cell-neuron interactions, and synaptic function in the hippocampus.^66^ Recently, the biological functions of SCFAs in the fetus have been well characterized. SCFAs are more than an energy source; they also serve as signaling molecules to regulate cellular functions via the activation of GPR41 and GPR43 (G-protein coupled receptors that sense SCFAs). In addition to SCFA production, the process of axonogenesis is promoted by maternal microbes through the modulation of metabolites (such as trimethylamine-N-oxide (TMAO) and imidazole propionate (IP)) during pregnancy, which could increase axon numbers and promote thalamocortical axonogenesis in the fetal brain.^61^ Furthermore, pathogen infection during pregnancy might negatively affect fetal brain development.^67^ Bacterial infection during pregnancy can increase the secretion of proinflammatory cytokine interleukin (IL)-17a, which can be transferred through the circulation, affect fetal brain development and induce an abnormal cortical phenotype.^68^ In rodent models, probiotic supplementation of the maternal diet during pregnancy promotes offspring brain development (Table 1). A deep understanding of the maternal microbiota and their metabolites that reach the fetus is crucial for facilitating clinical diagnoses and medical interventions. For instance, some metabolites (e.g., IL-17a) can be used as biomarkers for timely monitoring of fetal developmental brain abnormalities. Furthermore, other metabolites (e.g., SCFAs, TMAO, IP), which are beneficial to fetal brain development, could be used as maternal supplements to reduce neurodevelopmental disorders during pregnancy. Further research is needed to determine their clinical significance. In addition, it is crucial to assess the safety of these metabolites and to identify the optimal time for clinical intervention during pregnancy.

**Table 1.** Effect of maternal probiotic and prebiotic supplementation during pregnancy on brain development in offspring.

Probiotic and/or prebiotic	Time	Species	Impact on fetal brain development	Reference
Galacto-oligosaccharide (1.5 mg/ml)	During pregnancy and lactation	Mouse	Reduces expression of hippocampal glutamate receptor genes in offspring	^155^
Microbial metabolites	Gestational day 0.5 to day 14.5	Mouse	Supports fetal thalamocortical axonogenesis in the developing mouse offspring	^61^
*Lactobacillus* (10^9^ CFU)	During gestation	Mouse	Rescues brain region-specific microglial dysfunction of the male offspring	^161^
A combination of 1.5675 × 10 ^7^ CFU *Bifidobacteria* and 5.28 × 10^8^ CFU *Lactobacillus helveticus*	Embryonic day 0.5 to postnatal day 21	Mouse	Prevents parvalbumin positive (PV+) neuron loss, and the decrease in the γ-aminobutyric acid levels in the prefrontal cortex of adult offspring	^162^
A combination of *Lactobacillus rhamnosus* R0011 and *Lactobacillus helveticus* R0052 (10^9^ CFU/mL)	Postnatal day 2 to 14	Rat	Prevents precocious neural maturation in stressed infants	^163^
A combination of fructo- and xylooligosaccharides (3 g/kg/day)	During gestational days 0–19	Rat	Protects developing fetal brain against oxidative stress-mediated neurotoxicity	^164^
Oligofructose (10%)	During the 3rd week of pregnancy and throughout lactation	Rat	Prebiotics reverse maternal antibiotic use-induced decreased microglial response to LPS	^165^
A formulation containing *L. Fermentum 139, L. Fermentum 263* and *L. Fermentum 296* (ratio 1:1:1, 10^9^ CFU ml/L)	During pregnancy and lactation	Rat	Improves the sympathetic tone and autonomic dysfunction in male and female offspring	^166^
Inulin (2 g/kg/BW)	During gestational days 0–19	Rat	Reduces oxidative stress-mediated developmental origins of neurodegenerative disorders	^167^
Inulin (2 g/kg/day)	During pregnancy	Rat	Protects fetal brain from oxidative dysfunctions; protects developing brain against oxidative stress-mediated neurotoxicity	^168^

##### 2.3.2.2 Immune developmentt

The intestinal commensal microbiota is crucial for the establishment of the host’s innate and adaptive immunity. Recent evidence indicates that the maternal microbiome is involved in regulating the establishment of fetal immunity during pregnancy. Disruption of immunity might lead to allergic diseases, metabolic disorders, and infection in the fetus.^69^ A reversible germ-free colonization system was established to decipher the potential role of maternal microbes during pregnancy.^70^ With this system, transiently colonizing pregnant female mice with a live nonreplicating *E. coli* HA107 modulates the intestinal mucosal innate immune composition by increasing intestinal group 3 innate lymphoid cells and F4/80^+^CD11c^+^ mononuclear cells in their pups.^71^ These effects are partially dependent on maternal IgG that potentially retains and transmits bacteria-derived AhR ligands to offspring during pregnancy.^71^

Recently, more metabolites produced by maternal intestinal flora that regulate fetal innate immunity have been identified. Microbiota-derived SCFAs, which play multiple physiological and pharmacological roles, have been reported to boost Treg cell numbers.^72^ Specifically, bacteria-derived butyrate and propionate have been shown to increase the differentiation of extrathymic Treg cells in a CNS1-dependent manner in a mouse model.^72^ Acetate could prevent the development of preeclampsia through the promotion of fetal thymic and regulatory T-cell development.^73^ Furthermore, increased maternal dietary fiber intake during pregnancy has been reported to reduce asthma development in offspring through SCFAs, which might partially be due to the modulation of regulatory T lymphocytes in the fetal lung.^74^ In addition, the maternal microbiome regulates fetal immunity through the secretion of inflammatory factors. Maternal infection by a pathogen (*Yersinia pseudotuberculosis*) has been shown to regulate fetal immunity through an increase in the levels of interleukin (IL)-6, which crosses the placental barrier, promotes intestinal Th17 cell responses and enhances intestinal protective immunity in the fetus.^75^ Taken together, these findings suggest that maternal microbial metabolites (AhR ligands and SCFAs) and inflammatory factors (IL-6) synergistically regulate the early establishment of innate immunity before birth. It is worth noting that these identified microbial metabolites cannot fully explain the complicated molecular mechanism involved in gestational microbial shaping. Targeted and untargeted metabolomic studies are required to further identify other crucial microbial metabolites that participate in the regulation of fetal innate immunity.

To date, whether gestational colonization of the microbiota regulates adaptive immunity is unclear, which might be due to the controversy surrounding the prenatal microbiome. Amplification and activation of B and T cells are triggered mainly by bacterial colonization.^76,77^ Although colonization only during gestation does not affect adaptive immunity (populations of B or T cells) in infants,^78^ pregnant mothers who undergo antibiotic treatment have been shown to have lower proportions of both CD4^+^ and CD8^+^ T cells.^79–81^ Future research is needed to verify whether microbes are transferred from mother to fetus during the prenatal period, which could help clarify recent conflicting reports. Despite the inconsistent results regarding adaptive immunity regulation, it is worth noting that some preliminary evidence has indicated that probiotic supplementation of the maternal diet during pregnancy may reduce the risk of immune-mediated disease in infants (Table 2). Thus, modulation of the maternal diet, for instance, via direct supplementation with functional metabolites could be an efficient way to program the immunity of neonates.

**Table 2.** Effect of maternal probiotic and prebiotic supplementation during pregnancy on immune development in offspring.

Maternal diet	Time	Species	Impact on fetal immune function	Reference
10^9^ CFU of *Bifidobacterium lactis* and 10^9^ CFU of *Lactobacillus rhamnosus*	14 days prior to the cesarian section	Human	Protects the infant against detrimental long-term immunologic disturbances	^57^
Galacto-oligosaccharides and inulin in a 9:1 ratio, 4%	During acclimation, mating, and gestation	Mouse	Increases the presence of B and T regulatory immune cell subsets in the lymph nodes of offspring	^169^
Galacto-oligosaccharides and inulin (at a 9:1 ratio, 4%)	During mating and pregnancy	Mouse	Increases the abundance of regulatory B and T cells in the fetus	^170^
*Bifidobacterium breve M-16 V*	Pregnancy days 14 to the end of the lactation	Mouse	Prevents and/or alleviates allergic lung inflammation	^171^
Inulin (10%)	During pregnancy	Mouse	Regulates the differentiation of regulatory T-cell in offspring	^172^
Settings Lactobacillus fermentum CECT5716 (10^10^ UFC/day)	Pregnancy day 0 to lactation day 14	Rat	Increases IgA and IgG2a concentrations in offspring plasma	^173^
Sodium formate, sodium propionate, or sodium butyrate (250 mM)	Prior to pregnancy until day 40 after KRV infection	Rat	Downregulates virus-induced proinflammatory responses in the intestine; alters the B- and T-cell compartments in Peyer’s patches	^174^
Short-chain galacto- and long-chain fructo-oligosaccharides in a ratio of 9 : 1, 3%	From 2 weeks before mating until childbirth	Rat	Increases percentage of regulatory T cells and the concentrations of immunoglobulin (Ig)E and ovalbumin-specific IgG2a	^175^
*Bifidobacterium animalis subsp lactis BB-12*® (3 × 10^9^ CFU/ml) and *Propionibacterium jensenii 702* (8.0 × 10^8^ CFU/ml)	From 10 days before conception until postnatal day	Rat	Improves the plasma IgA and luminal IgA levels of stressed animals	^176^
Chitosan oligosaccharide (0.12/0.24 g/day)	During gestation and lactation	Pig	Increases the serum concentrations of IgM and sIgA in piglets	^177^
Mannan oligosaccharide (400 mg/kg)	From day 86 of gestation until weaning	Pig	Decreases concentrations of proinflammatory cytokines IL-2 and IL-4 in piglet serum	^178^
An insoluble/soluble fiber ratio of 3.89, 5.59, 9.12, and 12.81	During pregnancy	Pig	Improves antioxidative capacity and decrease the inflammatory response	^179^
Sugar beet pulp (20% SBP in gestation and 10% SBP in lactation)	Gestation and lactation	Pig	Ameliorates intestinal inflammation in piglets	^180^
Pregelatinized waxy maize starch plus guar gum (2.0%)	Pregnancy day 85 to end of lactation	Pig	Increases the plasma concentrations of IL-10 and TGF-β	^181^
Mannan oligosaccharide (400 mg/kg)	From d 86 of gestation until weaning	Pig	Increases the serum concentrations of IgA and IgG in piglets at weaning	^182^
Short-chain fructooligosaccharide (10 g sCFOS/d)	From last 4 weeks of gestation to the first 4 weeks of lactation	Pig	Improves the intestinal immune function of piglets	^183^
Seaweed extract (10 g/d)	Pregnancy day 107 to end of lactation	Pig	Increases piglet plasma IgG levels and improves intestinal inflammation	^184^

## 3. Maternal microbiome and neonatal health and development after birth

After birth, neonates are exposed to commensal microbes from a variety of sources. Maternal oral, gut and vaginal microbes all contribute to the initial bacterial inoculum in the newborn.^82^ Subsequently, antibiotic use, human milk, solid food, host genetics, and environmental exposure further affect the gut microbiome during early life.^83–85^ The complicated sources and transmission routes of pioneering microbes in infants make it difficult to systematically understand pioneer colonizers in newborns. In this section, we discuss the current understanding of the influence of the maternal microbiome derived from the maternal gut, vagina, and milk on early microbial colonization in neonates and the role of these microbes in neonatal health and development.

### 3.1 Maternal-source microbiome

#### 3.1.1 Maternal gut microbiome

The human maternal gut microbiota remains stable over the perinatal period (during late pregnancy and early lactation).^17^ To the best of our knowledge, change that occurs in the intestinal microbial community of humans during lactation is unknown. As a good model for the human microbiota, swine gut microbes harbor more Firmicutes (Lachnospiraceae, Christensenellaceae, *Clostridium*, *Ruminococcus*, and *Lactobacillus*) and Bacteroidetes (*Prevotella* and Paraprevotellaceae) during lactation than during gestation, with a corresponding augmentation in SCFA production.^86^ Similar to the situation that is observed during the gestational period, individual heterogeneity might also be a crucial factor that regulates the composition of the intestinal microbiome during lactation. The maternal gut microbiota regulates neonatal microbes via direct mother-neonate contact during vaginal delivery. In addition, *Bifidobacterium*, *Bacteroides*, *Parabacteroides* and members of Clostridia are shared among neonatal feces, maternal feces and breast milk,^87^ which indicates that the maternal gut microbiota can be transferred to the neonate through breast milk during lactation (this concept is discussed in the section on the maternal milk microbiome in detail).

#### 3.1.2 Vaginal microbiome

The vaginal microbiota during pregnancy is less diverse and comparatively stable throughout pregnancy.^88,89^ Five bacterial community state types (CSTs) have been defined in the vagina.^90^ In Asian and white women, CSTs are dominated by *Lactobacillus* species (*L. crispatus* (CST I), *L. gasseri* (CST II), *L. iners* (CST III) and *L. jensenii* (CST V)). CST IV (*Lactobacillus* spp.) is often observed in black and Hispanic women. This might partially explain why vaginally born infants initially exhibit an enrichment in *Lactobacillus* spp. in the oral cavity, skin and gut.^91^ During gestational progression, enriched *Lactobacillus* species might produce lactic acid and protect the vagina from bacterial vaginosis and pelvic inflammatory disease.^89,92^ Disruption of the vaginal microbiome during pregnancy is associated with preterm birth. *Lactobacillus iners* is correlated with the risk of preterm birth,^93^ while *Lactobacillus crispatus* dominance has a protective effect against preterm birth.^53^ Furthermore, preterm birth has been found to be associated with proinflammatory cytokines in vaginal fluid, including eotaxin, IL-1β, IL-6 and macrophage inflammatory protein (MIP)-1β.^94^

The mode of delivery (C-section-born infants vs. vaginally born infants) considerably affects microbiota colonization in newborns. C-section is usually conducted under antibiotic treatment to prevent infection, which further affects the transmission of maternal microbes to offspring. Thus, neonates born by C-section are associated with an aberrant and distinct gut microbiome that differs from that in vaginally born neonates.^95–98^ Intriguingly, microbes in vaginally born infants are affected mainly by maternal vaginal and fecal microbes, whereas microbes from C-section-born infants are more likely to be determined by maternal skin microbes.^91,99^ Infants born by C-section have a decreased abundance of *Bacteroides* spp. and *Lactobacillus*. ^100,101^ The shift in the microbial community caused by C-section might subsequently increase the risk of childhood sickness, such as asthma, obesity, allergies, and autoimmunity.^83,117,118^

Both vaginal seeding and maternal fecal microbiota transplantation can partially restore the microbiota difference caused by C-section delivery.^102^ However, unlike maternal intestinal strains, maternal vaginal strains seem to contribute to only a small and transient fraction of the microbes compared to the neonatal intestinal microbiota after birth. Oral administration of a maternal vaginal microbiota has a weak effect on the early development of the neonatal gut microbiome after one month.^103^ This might be because the vaginal environment is remarkably different from the gut environment, which makes the maternal vaginal strains difficult to seed. Currently, the understanding of neonatal microbiome seeding is limited, which prevents comprehensive assessment of the acute and lasting effects of the vaginal microbiome on newborns. Further research is needed to determine whether the maternal microbiota composition is optimal. Understanding the most effective combination of microbes and their metabolites may allow for the development of targeted probiotics that could be used for infants born by C-section.

#### 3.1.3 Milk microbiome

As the optimal nutrient source for infants, breast milk is the most important postnatal link between the mother and infant. In addition to macronutrients (protein, fat, and lactose), micronutrients (minerals and vitamins), oligosaccharides and immune factors, maternal milk contains a microbiota. Breast milk-derived microbes help neonates build a beneficial microbiome.^104^ The origins of bacteria in breast milk have not been fully elucidated, and these bacteria might be derived from maternal skin,^105^ the infant oral cavity,^106^ the peripheral environment,^107^ and the maternal gut.^108^ Currently, the neonatal oral cavity^109,110^ and maternal gastrointestinal tract^108,111^ are considered two major sources of milk microbes. Researchers have proposed “the enteromammary transfer hypothesis” and “the retrograde flow transfer hypothesis” to interpret the origin of the human milk microbiota (Figure 2).^112,113^

Both culture-based and sequence-based work suggest that streptococci and staphylococci are the most predominant taxa in human milk, but results regarding other bacterial taxa (such as lactobacilli) are inconsistent.^114^ To date, it has been difficult to conclude that the variation in milk microbes is attributed to geographical location or contamination during sampling.^115–117^ The milk microbiome is affected by lactation stage and influenced by maternal health status and BMI during pregnancy. In human milk, hindmilk (milk produced at the end of breastfeeding) has a higher bacterial load than foremilk (milk produced at the start of breastfeeding).^118^ Obese mothers have lower numbers of *Bifidobacterium* and higher numbers of *Staphylococcus* in their breast milk than normal-weight mothers.^119^ The effect of breast milk on infants lasts throughout the breastfeeding period. Approximately 33% and 23% of the bacterial taxa (especially *Streptococcus*, *Veillonella* and *Bifidobacterium* spp.) detected in infant feces are similar to those in maternal milk at 5 and 9 months, respectively.^118^

### 3.2 Maternal microbiome regulates neonatal health and development

#### 3.2.1 Early microbial colonization regulates neonatal health

Microorganisms in the neonatal gut regulate infant development and immune system maturation. There is a close relationship between intestinal microbial disorders and the development of pediatric diseases, such as obesity and metabolic disease.^120,121^ NEC and LOS are major causes of neonatal morbidity and mortality, and their occurrence is closely related to early microbial colonization. During early infancy, elevated levels of Enterobacteriaceae may contribute to the development of NEC.^122,123^ The intestinal microbiota in LOS infants has limited diversity, with low levels of *Bacteroides* and *Bifidobacterium* and a predominance of enterobacteria.^124^ In addition, early microbial colonization can affect asthma.^125^ The relative abundance of *Lachnospira*, *Veillonella*, *Faecalibacterium* and *Rothia* in the first 100 days of life has been found to be significantly reduced in children at risk of asthma.^125^ Furthermore, maternal group B S*treptococcus* colonization is related to increased aortic intima-media thickness in infants, which may have an impact on cardiovascular risk.^126^ Currently, it remains unclear how maternal microbes modulate pediatric disease through early microbial colonization. However, there is growing evidence indicating that the maternal microbiome plays an important role in the regulation of neonatal immunity and neurodevelopment.

#### 3.2.2 Maternal microbiome regulates neonatal development

##### 3.2.2.1 Immune development

**Figure 4.**
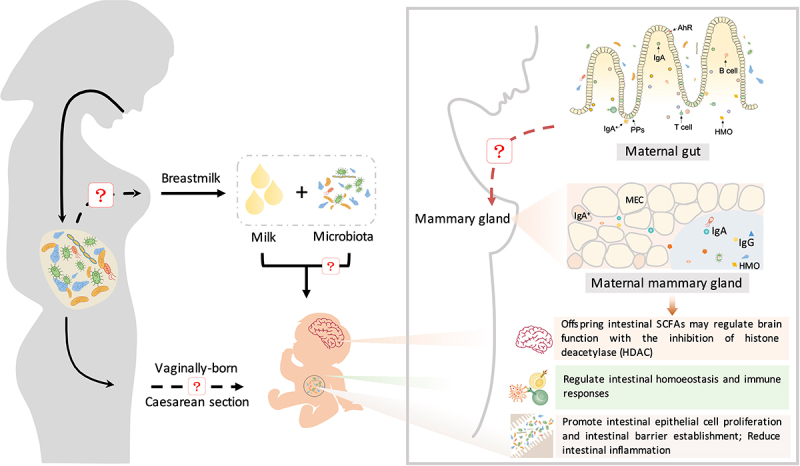
The maternal microbiota regulates neonatal immunity and brain development. Beneficial microbes inherited from breast milk contribute to the establishment of the immune system in neonates. Beneficial microbes from breast milk promote the production of immunoglobulins (IgA and IgG) and regulate intestinal homeostasis and immune responses (CD4+ T cells and monocytes) through the aryl hydrocarbon receptor (AhR). IgA^+^ plasma cells are recruited from maternal intestinal Peyer’s patches (PPs) and then translocate through the circulatory system to the mammary gland and secrete IgA. Furthermore, maternal intestinal Ig-coated bacteria can directly translocate to the mammary gland and then be enriched in maternal milk. Immunoglobulins in milk promote the differentiation of neonatal intestinal epithelial cells and the establishment of intestinal barrier function, reducing intestinal inflammation. During this process, immunoglobulins cooperate with gut microbes to regulate innate and adaptive immunity in neonates. In addition, maternal prebiotic intake increases the concentrations of offspring intestinal SCFAs, which may cross the blood – brain barrier and modulate brain function by inhibiting histone deacetylases (HDACs).

Beneficial microbes inherited from breast milk contribute to the establishment of the immune system in neonates, especially during the first 3 months of life (Figure 4).^127^ *Lactobacillus reuteri* enrichment in milk promotes the production of IgA through the induction of T cells and type 3 innate lymphocytes (ILC3s) in neonates.^127^ Milk-derived Bifidobacterium produces indolelactic acid via aromatic lactate dehydrogenase in infants.^128^ Importantly, indolelactic acid can further induce the immune responses of CD4^+^ T cells and monocytes through the activation of AhR and hydroxycarboxylic acid receptor 3.^128^ Human breast milk contains a large amount of indigestible oligosaccharides (human milk oligosaccharides, HMOs), which can be used by commensal organisms as well as probiotics.^129–131^ Bifidobacterium expressing HMO utilization genes can alleviate inflammatory responses by decreasing proinflammatory Th2 cells and promoting the expansion of anti-inflammatory regulatory T cells.^130^ Thus, maternal milk-derived HMOs and beneficial microbes can coordinately boost the immunity of neonates.

The maternal microbiome also regulates neonatal immunity through the secretion of milk immunoglobulin (Ig). Maternal milk, especially colostrum, is enriched in IgA. In contrast to other organic components in milk, Ig is not synthesized by mammary epithelial cells. In the mammary gland, both pIgR expression and IgA plasma cell accumulation are positively correlated with the production of milk IgA.^132^ Evidence suggests that milk IgA is either derived from serum or secreted from IgA^+^ plasma cells that translocate to the mammary gland.^133^ From pregnancy to the first few days of lactation, intestinal IgA^+^ plasma cells are significantly enriched, accompanied by a similar increase in the number of IgA^+^ plasma cells in the mammary gland.^134^ It is now clear that mammary gland IgA^+^ plasma cells are recruited mainly from intestinal Peyer’s patches (PPs) but not supramammary lymph nodes (LNs).^135^ Therefore, maternal gut microbes are involved in the regulation of milk IgA production.^135^ Maternal milk IgA production in germ-free (GF) mice is markedly lower than that in wild-type mice.^135^ *Bacteroides acidifaciens* and *Prevotella buccalis* cohabiting in the large intestine induce the synthesis of bacterium-specific IgA in milk.^135^

IgA binds to a variety of microbes, including four major phyla, namely, Firmicutes, Actinobacteria, Bacteroidetes, and Proteobacteria.^136^ Recently, accumulating evidence has revealed the potential role of IgA in the neonatal gut, which contributes to the establishment of the intestinal barrier and regulates the innate and adaptive immunity of neonates by binding to intestinal bacteria. First, IgA coats harmful bacteria and prevents their colonization and propagation. The enrichment of Proteobacteria, especially Enterobacteriaceae, is a crucial factor that triggers the development of NEC, an inflammatory gastrointestinal disease, in preterm infants.^8^ Milk IgA binds to Enterobacteriaceae and prevents the development of NEC in human infants.^137^ Second, IgA facilitates the adherence of beneficial bacteria (such as *Bacteroides fragilis*) to intestinal epithelial cells and promotes their proliferation.^138^ Third, IgA coating intestinal bacteria alleviates intestinal inflammation in neonates.^139^ The IgA coating of the microbiota decreases the proportions of ROR-γt^+^ Tregs and alleviates mucosal immune induction and inflammatory disease susceptibility.^140^ Furthermore, maternal intestinal Ig-coated bacteria can directly translocate to the mammary gland and then be enriched in maternal milk.^136^ IgA-coated Lachnospiraceae bacteria in milk can directly regulate the adaptive immune responses in the intestine.^141,142^ Due to technical issues, such as those with anti-Ig antibodies (monoclonal or polyclonal) and sequencing methods, it is difficult to identify the dominant Ig-coated community in maternal milk.^143–145^ In addition to IgA, IgG binds with bacteria and regulates gut immune function. Enteropathogenic *E. coli* (EPEC) is a foodborne pathogen that causes diarrhea in neonates.^146^ EPEC induces Ig production through “attaching and effacing” (A/E) lesions located on the intestinal epithelium.^147^ Interestingly, maternal pathogen-induced production of IgG but not IgA or IgM protects neonates against EPEC infection. Mechanistically, neutrophil phagocytosis is increased when pathogens are specifically coated with IgG.^148^ Although the origins of milk Igs and their capacity to prevent bacterial pathogenicity are well established, their role in promoting beneficial microbes and regulating immunity needs to be clarified. In addition, future research is required to identify other potential bacteria that can bind with Igs and their role in the regulation of neonatal gut immune function.

Allergic diseases affect the respiratory system, digestive system, skin and other systems. There are potential associations between immune dysfunction (such as an imbalance toward a T-helper-2 response and exaggerated IgE responses) and allergic diseases in infants.^149^ Atopic eczema is a strong predictor of allergic disease and occurs during childhood.^150^ During early infancy, a reduction in the abundance of bacteria with the ability to produce butyrate and propionate was observed in infants with atopic eczema.^151^ The World Allergy Organization (WAO) guideline panel suggests the use of probiotics in pregnant and breasting women with children at high risk for allergies.^152^ This recommendation could efficiently prevent the development of atopic eczema during childhood. Additionally, initial microbial colonization is associated with other immune-mediated diseases, such as inflammatory bowel disease (IBD)^153^ and asthma.^154^ More research is needed to clarify whether and how maternal microbes modulate clinical disease.

##### 3.2.2.2 Brain development

Recent evidence also indicates that the maternal microbiome regulates neonatal brain development (Figure 4). Maternal prebiotic intake reduces anxiety and promotes brain development. The intestinal microbiome composition in the offspring of mice fed prebiotics differs from that in control offspring, with significant decreases in the abundances of *Bacteroides caecimuris, the Barnesiella* genus, and the *Bifidobacterium* genus.^155^ Importantly, postnatal maternal prebiotic intake significantly increases the concentrations of intestinal SCFAs in offspring, which might cross the blood – brain barrier and regulate brain function via the inhibition of histone deacetylase (HDAC).^155^ In contrast, maternal infection with *E. coli* O16:H48 has a negative effect on the growth of neonates in a mouse model.^156^ *E. coli* O16:H48 can disrupt the production of precursors of neurotransmitters (such as serotonin), which negatively affects maternal behavior.^156^ Poor maternal behavior blocks milk availability for neonates and subsequently affects the brain development of neonates.^156^

During pregnancy, the developing fetus is entirely dependent on maternally derived nutrients. However, during lactation, when maternal breast milk is insufficient, an alternative form of enteral nutrition for preterm or low birth weight (LBW) infants is artificial formula. Currently, artificial formula contains more nutrients than breast milk but might be deficient in some nonnutritive bioactive components. Fortification of artificial formulas with microbiota and functional metabolites such as milk-derived Bifidobacterium and *Lactobacillus reuteri* as well as indolelactic acid could be beneficial for preterm or LBW infants and might have great potential for alleviating clinical diseases such as IBD and asthma.

## 4. Conclusion and perspectives

A healthy maternal microbiota is required for the health and development of offspring. During the progression of gestation and lactation, the maternal gut microbe composition is comparatively stable. Individual heterogeneity is the main factor that shapes the maternal intestinal microbiome. These microbes do not act as silent passengers but have long-term effects that reduce the risk of immune-mediated illness and improve the cognitive outcomes of offspring. During pregnancy, maternal microbial metabolites can be transported from the placenta to the fetus to regulate immunity and recognition. Whether a small number of bacteria exist in the fetus before birth is uncertain, and further research is needed.

Microbial colonization of neonates begins at birth. The transfer of microbes from mother to newborn is influenced by many factors, such as birth route and nursing. Vaginal birth, breastfeeding, and skin-to-skin care are considered predominant factors in establishing the close relationship between the mother and infant microbiome. Maternal microbes that originate from the maternal gut but not the vagina contribute mainly to the early colonization of the microbiome. Maternal milk is rich in microbes, oligosaccharides and Igs, which boost immune and brain development. The disruption of maternal-to-child microbiome transfer might lead to short- and/or long-term adverse health issues.

Recently, great interest in modulating the maternal and neonatal microbiomes through therapeutic manipulation has emerged. Maternal probiotic and prebiotic supplementation during pregnancy have been found to influence the regulation of offspring brain and immune development (as shown in Tables 1 and 2). The analysis and modulation of specific bacterial metabolites, such as IL-17a, AhR ligands and SCFAs, may provide a noninvasive monitoring and intervention strategy to improve adverse prognoses and alleviate clinical symptoms. Further studies are needed to identify other crucial microbial metabolites that participate in regulating fetal immune and brain development using both targeted and untargeted metabolomics.

### Outstanding questions

Numerous outstanding questions that deserve to be addressed in the future are listed below.

1. Is the womb sterile or not?
2. Are there key molecular, genetic or microbiome-based biomarkers available to test the “gut-placental transmission hypothesis”, “entero-mammary transfer hypothesis” and “retrograde flow transfer hypothesis”?
3. Which maternal-derived microbes participate in early colonization of the offspring’s gut and what is the potential role of maternal-derived microbes in later microbiota development?
4. In the milk, are there any functional metabolites synthesized locally in the mammary gland?
5. Could diet-derived maternal microbial metabolites in milk be modulated by dietary intervention or by direct supplementation with targeted metabolites?

Box 1. Is the womb sterile?

To verify this hypothesis, pregnant mice were inoculated with genetically labeled *Enterococcus faecium*.^157^ Notably, genetically labeled *Enterococcus faecium* was detected in the meconium of fetuses delivered via sterile C-section, which provides primary evidence for maternal microbial transmission to the fetus.^157^ However, recently, inconsistent results regarding in utero bacterial colonization among different studies indicate that the detection of a prenatal microbiome might just be due to contamination of reagents, sample handling, etc.^158^ Furthermore, even though many studies have reported that bacterial DNA can be detected in first-pass meconium samples, most of the samples are collected hours to days after birth.^159^ Meconium collected during cesarean deliveries indicates that healthy infants do not harbor a detectable gut microbiota.^159^ Thus, the microbiotas previously identified in neonatal meconium might be acquired during and/or after birth. In addition, bacterial DNA does not indicate the existence of live microbes.

Recent evidence supports the ‘sterile womb’ hypothesis, as the existence of C-section-born germ-free animals strongly argues against the existence of microbes in the placenta. However, an intense debate about the existence of microbes in the fetus is ongoing. Recently, although strict contamination controls (environmental control, operator control, PBS buffer control, and reagent control) were carefully applied in an experiment,^160^ weak but consistent microbial signals were still detected by 16S rRNA sequencing in fetal organs (including the gut, skin, placenta, and lungs) during the 2^nd^ trimester of gestation. More importantly, these microbes were viable and could be cultured and propagated *in vitro*. These sparsely distributed microbes, such as *Staphylococcus* and *Lactobacillus*, induce the activation of memory T cells, which might contribute to the establishment of early immunity.^160^ Currently, early fetal microbial colonization is thought to be affected by microbes from the maternal vagina or gut. The contradictions in the results regarding the sterile womb necessitate the collection and assessment of additional evidence in the future.
